# The emotional side of post-traumatic stress reaction during COVID-19 pandemic: an Italian survey

**DOI:** 10.1186/s12889-022-12749-1

**Published:** 2022-02-16

**Authors:** Gianluigi Ferrante, Pierre Gilbert Rossini, Stefano Rousset, Luca Ostacoli, Cristiano Piccinelli, Sara Carletto, Livia Giordano

**Affiliations:** 1 SSD Epidemiologia Screening, CPO-AOU Città Della Salute E Della Scienza Di Torino, Via Cavour, 31, 10123 Torino, Italy; 2 Department of Clinical and Biological Sciences, University of Torino, Torino, Italy; 3 Department of Neuroscience, University of Torino, Torino, Italy

**Keywords:** COVID-19, Lifestyle, Mental health

## Abstract

**Background:**

Social restrictions due to COVID-19 might have had a significant impact on mental health. The aim of this study was to assess the prevalence of four emotional domains (nervousness, anger, numbness, physiological arousal) in a sample of citizens during the first pandemic wave in 2020, and their association with sociodemographic characteristics, housing conditions and lifestyle modifications.

**Methods:**

A cross-sectional study based on a self-administered online questionnaire was set up to investigate emotions. Respondents were recruited through a non-probabilistic snowball sampling approach. The SPAN questionnaire was used to measure emotions in the interviewed population. The association between emotions and independent variables (gender, age, marital status, educational level, working condition, housing condition, COVID-19 positivity, sleep disturbance, increase in alcohol consumption and decrease in physical activity) was assessed through the multivariate Poisson regression.

**Results:**

A total of 6,675 subjects were included in the analysis. Almost half of respondents (48.9%) experienced nervousness, 41.3% anger, 15.6% numbness and 18.8% physiological arousal. Females were more likely to face nervousness, anger and physiological arousal. For all the outcomes a decreasing trend was observed from younger to older. Singles were more likely to experience numbness compared to married people. Increase in alcohol consumption was associated with nervousness, anger and numbness. Decrease in physical activity was associated with nervousness, anger and physiological arousal. Restless sleep was the variable most associated with all emotional domains.

**Conclusions:**

The first COVID-19 pandemic wave had a significant emotional impact on this sample, especially among younger people, singles and females. Even without reaching clinical relevance, these emotions could represent a form of psychological distress, which requires the implementation of preventive strategies, in particular regarding lifestyle care.

**Supplementary Information:**

The online version contains supplementary material available at 10.1186/s12889-022-12749-1.

## Background

In 2019 a novel coronavirus (SARS-COV-2) appeared in China and spread globally. The second largely affected country was Italy, starting from February 2020, especially in the northern regions [1]. From March 2020 to the present, the Italian Government implemented several restrictive measures to reduce the viral transmission. The first lockdown was national and entailed strict limitations to the public, economic and social life. This may be critical to consider, given that there are potentially millions of people struggling with the isolation involved in the quarantine and with the emotional response triggered by the pandemic. Indeed, the global prevalence of depressive symptoms stands now at 25%, which is 7 times higher than the same recorded in 2017 (3.44%) [2] and USA prevalence of depression is 3 times more than the pre COVID-19 era [3]. Nevertheless, the psychological impact of COVID-19 has not been fully outlined, since evidence showed this is both significant [4, 5] and small [6, 7]. However, divergent findings could be explained by the use of different instruments to detect psychological distress and by the inclusion of subthreshold symptoms or of full-blown clinical presentations. Indeed, it has been suggested that this pandemic may operate as a traumatic stressor, beyond its clinical displays, leading to various acute and negative emotional responses [8]. Following this idea, it appears useful to describe the psychological sequelae of this pandemic in terms of emotions, rather than clinically relevant symptoms, which can be underestimated for different reasons (i.e. small samples, methodological differences, under-expression for fear of stigma). A recent review [7], reported as common negative reactions to the COVID-19 pandemic, a nonspecific fear of contagion, pervasive anxiety, frustration, boredom and disabling loneliness. This picture is confirmed by another review where high rates of Post-traumatic Stress Disorder (PTSD) clinical features in response to quarantine, such as hypervigilance, chronic alert, hyperarousal, confusion and anger, have been found, even for similar coronaviruses, as SARS [9]. Moreover, data from Internet-related services seem to point in the same direction, highlighting the expression of negative sentiments [10–12]. An anonymous survey was carried out in spring 2020 by the Centre for Epidemiology and Cancer Prevention in Piedmont (CPO Piemonte) to explore emotional experience and lifestyle habits in a sample of Italian citizens during the first COVID-19 pandemic wave [13].

The aim of the present study was to explore the prevalence of the four emotional domains (nervousness, anger, numbness, physiological arousal) and their association with sociodemographic characteristics, housing conditions and lifestyle modifications.

## Methods

Between April 21^st^ and June 7^th^ 2020, CPO Piemonte conducted a cross-sectional study based on an anonymous self-administered online questionnaire aiming at investigating emotional experience and lifestyle habits during the lockdown period.

Respondents were recruited using a non-probabilistic snowball sampling approach: the weblink to the questionnaire was disseminated through institutional websites, messaging apps and institutional/private social networks accounts. The questionnaire was accessible from smartphones, tablets and personal computers. It is divided into 7 sections: i) socio-demographic features and housing conditions; ii) information on employment; iii) physical activity (PA) in leisure time; iv) eating habits and anthropometric data; v) tobacco smoking habits; vi) state of health; vii) mental well-being and sleeping disorders. No question was mandatory (Additional file 1).

Educational level has been grouped into two categories, low (none/elementary school or Junior high school), and high (High school or University).

The definition of overcrowded house was based on the combination of the number of household members and number of premises in the house. If less than one premise per person was available, the dwelling was considered overcrowded.

The employment categories have been combined into three groups according to the working condition: students, workers (self-employed, employees, housewives and unemployed) and pensioners.

Leisure time PA was investigated asking for the change since the lockdown came into effect. A dichotomous variable for identifying a reduction in PA was constructed: yes (“yes, I reduced it”), no (“no, I did not reduce” or “yes, I increased”). Similarly, a dichotomous variable was created to identify an increase in alcohol consumption: yes ("yes, I increased it"), no ("it remained unchanged" or “no, I have decreased it”).

People were defined as Covid-19 positive either if they tested positive to a molecular swab or were recommended by the General Practitioner (GP) to remain isolated due to a possible Covid-19 infection.

Sleep disturbance was evaluated asking the respondents if he/she had experienced restless or disturbed sleep in the past two weeks. A dichotomous variable was then constructed: no (“not at all”), yes (“a little” or “moderately” or “a lot” or “very much”).To assess post-traumatic emotional reactions, the four-item SPAN (Startle, Physiological Arousal, Anger, Numbness) instrument, derived from the DTS (Davidson Trauma Scales) was adopted [14, 15]. The SPAN questionnaire explores four emotional domains in the short term: nervousness (in the past two weeks have you felt nervous or easily frightened?); anger (in the past two weeks have you felt irritable or have you had outbursts of anger?); numbness (in the past two weeks have you felt unable to experience feelings of sadness or affection?); physiological arousal (in the past two weeks have you had any physical disturbances related to thoughts on the ongoing emergency, such as sweating, tremors, rapid heartbeat, shortness of breath, nausea, or diarrhoea?). For each of the four SPAN items, the questionnaire requires to report the frequency (“never”, “only once”, “2–3 times”, “4 or more times, almost every day”) and the intensity (“minimal”, “moderate”, “high marked”, “extreme discomfort”) of the emotion investigated. Subsequently, a score ranging from 0 to 4 is constructed. The score is 0 if the frequency value is "never". The score is 1, 2, 3 or 4 if the frequency value is other than “never” and the corresponding intensity value is "minimal", "moderate", "high marked" or "extreme discomfort" respectively. A dichotomous variable for each item is then constructed to create the outcome indicators according to the score obtained: nervousness, anger, numbness and physiological arousal. If the score ranges between 0 and 1 the emotion is considered absent; if the score ranges between 2 and 4 the emotion is considered to be present.

Descriptive statistics are presented through absolute numbers and percentages for categorical variables, and Chi-squared tests are used to evaluate differences in proportions. Multivariate Poisson regression models with robust variance estimation are used to investigate the association between emotions and socio-demographic characteristics, housing conditions, occupation, positivity to Covid-19 and sleep disorders. Prevalence ratios adjusted (AdjPR) for gender, age, marital status, educational level, occupation, overcrowded living environment, availability of an external space, living alone, positivity to Covid-19 and restless sleep were calculated along with respective p-values. Statistical significance was set at alpha = 0.05. All the analyses were conducted through Stata15.1 statistical software.

## Results

A total of 10,758 persons participated in the survey, but only 7,487 (72.9%) reported a minimum set of information, characterizing their socio-demographic profile (i.e. age, gender and province of residence). Six subjects less than 16 years old were excluded from the analysis since it was not possible to obtain the informed consent from parents or legally authorized representatives. Among the remaining respondents, 6,675 subjects (62%) completed the SPAN questionnaire and represent our study population.

Most of the questionnaires (87%) came back before May 3^rd^ 2020, the period with strict isolation measures. Among the 6,675 subjects of the study population, 92% lived in Northern Italy, 71.5% were female, the mean age was 48.7 years and the most represented age classes were 30–49 (39.9%) and 50–69 years (45%).

The majority were Italian (96.9%), workers (82.2%), married (66.8%) with a high educational level (93.4%). Only 3.2% lived in an overcrowded dwelling and 73.3% in a house with an external space. One out of ten (10.8%) increased alcohol consumption and 56.3% reduced the time spent for PA (Table 1).

**Table 1:** Distribution of sociodemographic characteristics, housing conditions and lifestyles modifications of respondents, overall and by gender

		**Overall**		**Males**		**Females**		
		n	%	n	%	n	%	*p*-chi2
***Total***								
** Age group**	16–29 years	645	9.7	225	11.8	420	8.8	< 0.001
	30–49 years	2664	39.9	726	38.2	1938	40.6	
	50–69 years	3007	45.0	815	42.8	2192	45.9	
	70 + years	359	5.4	136	7.2	223	4.7	
** Working condition**	Student	290	4.4	99	5.2	191	4.0	0.066
	Worker	5447	82.2	1524	81.0	3923	82.7	
	Retired	887	13.4	259	13.8	628	13.2	
** Marital status**	Married	4377	66.8	1215	64.9	3162	67.6	< 0.001
	Single	1382	21.1	497	26.5	885	18.9	
	Separated	625	9.5	138	7.4	487	10.4	
	Widowed	168	2.6	23	1.2	145	3.1	
** Educational level**	High (high school/university)	6113	93.4	1759	94.3	4354	92.9	< 0.001
	Low (none/elementary school or Junior high school)	433	6.6	105	5.7	328	7.1	
** Nationality**	Italian	6274	96.9	1804	97.6	4470	96.6	0.045
	Foreign	202	3.1	45	2.4	157	3.4	
** Overcrowded house**	No	6232	96.8	1781	97.0	4451	96.8	0.615
	Yes	204	3.2	55	3.0	149	3.2	
** Household members**	3 or more people	3815	58.2	1054	56.3	2761	59.0	0.043
	2 people	1772	27.0	510	27.3	1262	26.9	
	1 person	969	14.8	307	16.4	662	14.1	
** External space**	No	1724	26.7	545	29.4	1179	25.5	0.002
	Yes	4746	73.3	1309	70.6	3437	74.5	
** Town of residence size**	Less than 10.000 inhab	1860	28.5	459	24.5	1401	30.2	< 0.001
	Between 10.000 and 100.000 inhab	2414	37.0	689	36.7	1725	37.1	
	Over 100.000 inhab	2247	34.5	729	38.8	1518	32.7	
** Geographical area of residence**	Northern Italy	6119	91.7	1672	87.9	4447	93.2	< 0.001
	Central Italy	259	3.9	106	5.6	153	3.2	
	Southern Italy and Islands	297	4.4	124	6.5	173	3.6	
** Healtcare worker**	No	5052	77.3	1541	81.9	3511	75.4	< 0.001
	Yes	1487	22.7	340	18.1	1147	24.6	
** COVID-19 positive**	No	6031	93.5	1744	93.8	4287	93.3	0.483
	Yes	421	6.5	115	6.2	306	6.7	
** Increase in alcohol consumption**	No	4729	89.2	1208	85.4	3521	90.6	< 0.001
	Yes	570	10.8	206	14.6	364	9.4	
** Decrease in physical activity**	No	2768	43.7	783	42.5	1985	44.1	0.257
	Yes	3572	56.3	1057	57.5	2515	55.9	

**Fig. 1:**
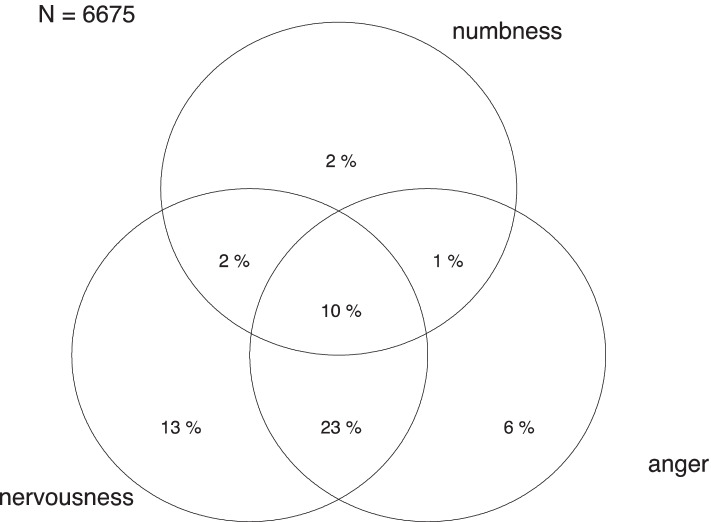
Emotions overlap

During the strict lockdown period, 48.9% of participants experienced nervousness, 41.3% anger, 15.6% numbness and 18.8% physiological arousal (Tables 2 and 3). The two most frequent combinations were nervousness and anger (23%) and nervousness, anger and numbness (10%) (Fig. 1).

**Table 2:** Association of demographic characteristics, housing conditions and lifestyles modifications with nervousness and anger

		**Nervousness**			**Anger**		
		%	adj PR	IC	%	adj PR	IC
	***Total***	***48.9%***			***41.3%***		
**Gender**	Male	37.4%	1		34.4%	1	
	Female	53.5%	1.33	1.21—1.48	44.0%	1.19	1.07—1.32
**Age group**	16–29 years	60.1%	1		53.0%	1	
	30–49 years	55.9%	0.9	0.77—1.06	49.1%	0.96	0.8—1.15
	50–69 years	42.3%	0.71	0.59—0.85	33.3%	0.73	0.6—0.89
	70 + years	32.0%	0.6	0.43—0.84	27.6%	0.74	0.51—1.06
**Marital status**	Married	47.2%	1		40.9%	1	
	Single	54.3%	1.03	0.9—1.17	46.0%	1.01	0.88—1.16
	Separated	49.6%	1.03	0.89—1.21	35.6%	0.91	0.77—1.09
	Widowed	46.6%	1.07	0.79—1.46	30.3%	0.88	0.61—1.28
**Educational level**	High	49.2%	1		41.4%	1	
	Low	45.5%	1.01	0.84—1.21	38.8%	0.99	0.82—1.21
**Working condition**	Student	60.4%	1		58.4%	1	
	Worker	50.2%	0.98	0.79—1.22	42.6%	0.84	0.67—1.05
	Retired	36.7%	0.9	0.69—1.21	27.6%	0.7	0.51—0.94
**Overcrowded house**	No	48.6%	1		41.0%	1	
	Yes	61.1%	1.1	0.89—1.35	53.8%	1.05	0.84—1.32
**External space**	No	54.0%	1		44.7%	1	
	Yes	47.2%	0.92	0.84—1	40.1%	0.95	0.86—1.04
**Living alone**	No	48.9%	1		42.2%	1	
	Yes	49.0%	1.01	0.87—1.16	35.8%	0.89	0.76—1.05
**COVID-19 positive**	No	48.7%	1		41.0%	1	
	Yes	54.6%	0.97	0.83—1.14	47.8%	1.01	0.84—1.19
**Restless sleep**	No	18.8%	1		18.8%	1	
	Yes	56.9%	2.77	2.39—3.2	47.1%	2.27	1.95—2.64
**Increase in alcohol consumption**	No	49.1%	1		41.7%	1	
	Yes	61.6%	1.16	1.03—1.31	55.6%	1.23	1.08—1.4
**Decrease in physical activity**	No	45.9%	1		38.9%	1	
	Yes	51.5%	1.12	1.03—1.22	43.6%	1.14	1.04—1.25

Being female was significantly associated with nervousness (53.5% vs 37.4%; AdjPR 1.33, 95% CI: 1.21—1.48), anger (44% vs 34.4%; AdjPR 1.19, 95% CI: 1.07—1.32) and physiological arousal (21.5% vs 12%; AdjPR 1.53, 95% CI: 1.29—1.8). Numbness prevalence was similar between the sexes (15.8% vs 15.1%; AdjPR 1, 95% CI: 0.85—1.18) (Table 2, Table 3).

**Table 3:** Association of demographic characteristics, housing conditions and lifestyles modifications with numbness and physiological arousal

		**Numbness**			**Physiological arousal**		
		%	adj PR	IC	%	adj PR	IC
	***Total***	***15.6%***			**18.8%**		
**Gender**	Male	15.1%	1		12.0%	1	
	Female	15.8%	1	0.85—1.18	21.5%	1.53	1.29—1.8
**Age group**	16–29 years	26.1%	1		21.4%	1	
	30–49 years	16.6%	0.7	0.54—0.9	20.7%	0.91	0.7—1.19
	50–69 years	13.0%	0.57	0.43—0.77	17.4%	0.86	0.65—1.14
	70 + years	11.0%	0.47	0.26—0.83	10.9%	0.61	0.34—1.1
**Marital status**	Married	13.5%	1		17.5%	1	
	Single	21.9%	1.26	1.01—1.57	21.3%	1.25	1.02—1.54
	Separated	16.3%	1.09	0.82—1.44	23.0%	1.31	1.04—1.64
	Widowed	14.9%	1.02	0.58—1.8	16.6%	0.96	0.56—1.64
**Educational level**	High	15.6%	1		18.5%	1	
	Low	16.6%	1.15	0.85—1.57	22.9%	1.18	0.9—1.56
**Working condition**	Student	26.8%	1		20.8%	1	
	Worker	15.6%	0.98	0.7—1.36	19.8%	1.21	0.84—1.75
	Retired	11.7%	0.98	0.62—1.55	12.2%	0.9	0.56—1.44
**Overcrowded house**	No	15.6%	1		18.5%	1	
	Yes	17.5%	0.97	0.66—1.42	26.7%	1.17	0.84—1.62
**External space**	No	18.5%	1		21.6%	1	
	Yes	14.7%	0.85	0.73—0.99	17.7%	0.89	0.77—1.03
**Living alone**	No	15.1%	1		18.7%	1	
	Yes	18.4%	1.08	0.85—1.37	19.5%	0.96	0.7—1.19
**COVID-19 positive**	No	15.2%	1		18.1%	1	
	Yes	19.1%	1.03	0.77—1.36	29.5%	1.25	0.99—1.57
**Restless sleep**	No	6.4%	1		4.3%	1	
	Yes	18.2%	2.73	2.1—3.5	22.7%	4.24	3.13—5.68
**Increase in alcohol consumption**	No	15.7%	1		19.6%	1	
	Yes	22.4%	1.33	1.08—1.63	20.7%	0.97	0.79—1.19
**Decrease in physical activity**	No	14.6%	1		17.1%	1	
	Yes	16.3%	1.15	0.99—1.34	20.1%	1.2	1.05—1.37

A decreasing trend was observed across age groups, from younger to older people, for each outcome. In particular, older people (> 70 years) compared to younger ones (16–29 years) less frequently reported nervousness (32% vs 60.1%; AdjPR 0.6, 95% CI: 0.43—0.84) and numbness (11% vs 26.1%; AdjPR 0.47, 95% CI: 0.26—0.83), while being retired compared with being a student was associated with anger (27.6% vs 58.4%; AdjPR 0.7, 95% CI: 0.51—0.94) (Table 2, Table 3).

Compared to married people, singles were more likely to experience numbness (21.9% vs 13.5%; AdjPR 1.26**, **95% CI: 1.01—1.57) and physiological arousal (21.3% vs 17.5%; AdjPR 1.25, 95% CI: 1.02—1.54), while being divorced was significantly associated with physiological arousal (23% vs 17.5%; AdjPR 1.31, 95% CI: 1.04—1.64) (Table 2, Table 3).

The presence of an external space in the house was associated with a lower percentage of people reporting nervousness (47.2% vs 54%; AdjPR 0.92, 95% CI: 0.84 – 1.00) and numbness (14.7% vs 18.5%; AdjPR 0.85, 95% CI: 0.73 – 0.99) (Table 2, Table 3).

Having experienced restless sleep was strongly associated with all emotions: nervousness (56.9% vs 18.8%; AdjPR 2.77, 95% CI: 2.39—3.2), anger (47.1% vs 18.8%; AdjPR 2.27, 95% CI: 1.95—2.64), numbness (18.2% vs 6.4%; AdjPR 2.7, 95% CI: 2.1—3.5) and physiological arousal (22.7% vs 4.3%; AdjPR 4.24, 95% CI: 3.13—5.68) (Table 2, Table 3).

Respondents who increased alcohol consumption were more likely to experience nervousness (61.6% vs 49.1%, AdjPR 1.16, 95% CI: 1.03—1.31), anger (55.6% vs 41.7%, AdjPR 1.23, 95% CI: 1.08—1.4) and numbness (22.4% vs 15.7%, AdjPR 1.33, 95% CI: 1.08—1.63), while the decrease in physical activity was associated with nervousness (51.5% vs 45.9%, AdjPR 1.12, 95% CI: 1.03—1.22), anger (43.6% vs 38.9%, AdjPR 1.14, 95% CI: 1.04—1.25) and physiological arousal (20.1% vs 17.1%, AdjPR 1.2, 95% CI: 1.05—1.37) (Table 2, Table 3).

## Discussion

To our knowledge, this is the first work providing a description of the psychological response to COVID-19 in terms of emotions, rather than clinical symptoms, using the SPAN scale. In this regard, it is worthy to note that the online survey investigated the emotional reaction to the limitation induced by the pandemic state. Among the emotional sequelae of the pandemic considered as outcomes indicators, the most frequent combination was nervousness and anger (23%). Seen in light of the model proposed by Henry Selye [16], these may be understood as part of the acute phase of a triphasic physiological stress reaction: the *alarm* reaction (i.e. sympathetic activation of the nervous system and physiological arousal increase) is followed by a *resistance* phase and by the possible *exhaustion* stage, where the body and the mind health may be threatened and where the clinical relevance can be reached [17]. Nervousness and anger may be seen as the physiological strain of the individual to cope with the first national lockdown, as these emotions are coherent with the *alarm* reaction, with no necessary clinical relevance: this could explain why in several studies the psychological impact of the pandemic has been indicated as moderate [6, 7]. Moreover, alongside with the extension of the lockdown (October—December 2020/February—April 2021) manifestations of *exhaustion* are expected to be found, together with emotions such as numbness and sadness. This reflects the distribution of emotional reactions that have been found in our sample, the majority of which (92%) was interviewed before the 3rd of May 2020: indeed, the 15,6% of the subjects only experienced numbness, compared to the 48.9% and the 41.3% that experienced respectively nervousness and anger. However, this needs more longitudinal studies to be confirmed [18]. Furthermore, to better characterize the emotional response to COVID-19, the analysis highlighted potential factors associated with the occurrence of negative feelings during the lockdown period, which are going to be discussed in the following section.

### Being female

The association between female gender and greater level of psychological distress has been reported in several previous studies on the psychological impact of the COVID-19 pandemic [18]. Furthermore, epidemiological studies reported higher prevalence of depression, anxiety and PTSD in women [19]. This may be consistent with the Italian context, where women are burdened by their *double role* of housekeepers, family caregivers and workers [20]. Higher levels of nervousness, anger and physiological arousal, may reflect the combination of the pandemic stressor together with the one coming from the social platform, which demands women to be even more resilient in different settings, such as the family and the workplace environment.

### Being young

Our results suggest that older people (> 70 years) tend to be less nervous, angry and numb and to experience less physiological symptoms, compared to younger people (16–29 years). The elderly seems more resilient than younger people, confirming evidence from other studies [21, 22]. In contrast, adolescents and young adults reported higher levels of nervousness, anger and, to a lesser extent, of numbness. This may fit with the evidence that the psychological impact of COVID 19 on the younger people resulted in significant emotional changes [23].

An updated definition of adolescence has been stretched ahead over time [24]: the *new adolescence* is considered to go from 10 to 25 years, and this is particularly true in the Italian context, where the transition from school to an independent work is slower [25]. Based on this, it may be possible that adolescents and young adults share similar psychological mechanisms in reaction to the actual pandemic, which are different from those displayed by older people. In particular, a lower risk perception and a greater rules’ aversion, features of the adolescent brain [26], together with a low mortality rate of COVID-19 in young people [27], may make them less compliant with the restrictions. Moreover, nervousness and anger may be worsened by the limitation of freedom and social life that those rules entail, including the school closures [28]. This may be because the ability to regulate their own emotions is, in younger people, much more dependent on the developmental environment than that in adulthood [29]. For these reasons, this seems a critical population to support, throughout the unfolding of the COVID-19 pandemic.

### Being single or separated

This is a previously reported risk factor in developing psychological distress and depressive symptoms [22]. Nevertheless, it seems possible not to consider singlehood as a risk factor per se, but in the context of an imposed social distancing, where any kind of relationship or human contact is forbidden, excluding those with relatives or partners. In this regard, there is a lack of data about the effect of social distancing on mental health outcomes, even if it may increase fear, anxiety symptoms, loneliness, and depressed mood [30].

### Increasing the intake of alcohol

The consumption of alcohol has been shown to rise during the pandemic [31], since this may represent one of the possible coping strategies to deal with negative emotions [32]. Indeed, both in clinical and general samples, it has been shown that the implementation of avoidant coping strategies is positively correlated with drinking behaviour and may account for its maladaptive use [33]. Finally, previous studies [34] showed the association between a variety of psychiatric disorders and the alcohol abuse level, where anger and nervousness could be more implicated.

### Reducing the physical activity

The current pandemic greatly limited the possibility to practice physical activity, which has several benefits on mental health outcomes, both at cognitive and emotional level [35]. Indeed, it has been reported that physical activity can improve psychological well-being [36], even during the COVID-19 pandemic, where exercising softened the virus' impact on anxiety and depression levels [37]. Therefore, it may not be surprising that our results associated a decreased physical activity with increased negative emotions, such as anger, nervousness and increased physiological arousal.

### Experiencing sleep disturbances

Sleep patterns have been disrupted by the pandemic, given that this issue globally affected the 40% of people from the general and the healthcare population [38]. To preserve a good sleep quality during COVID-19 pandemic is relevant, given the multiple levels that its deprivation can affect: indeed, it has been reported that the lack of sleep can impact the cognitive performances, the circadian rhythms, the immune activity, the emotional regulation and the sympathetic nervous system activity, leading to an increased stress responsivity [39]. Interestingly, in our study restless sleep was the variable most strongly associated with all emotional domains, suggesting the same pivotal role of sleep in regulating affective expression and well-being.

### Housing conditions

Among housing conditions, the presence of an external space is slightly associated with less numbness. This is consistent with previous reported data from the Italian context [40], where a strong association between depressive symptoms and poor housing has been shown. In particular, it seems that safe, open and natural housing spaces can strongly impact the quarantined quality and mental health, reducing the sensation of being trapped [41], even if further studies are needed to draw firmer conclusions.

### Limitations and strengths

This study has few limitations. Firstly, as a snowball sampling was used, the sample should not be considered representative of the Italian general population.

A selection bias might have occurred, especially in the older population, composed of highly educated individuals. This potential bias could have influenced one of the findings of the survey, namely a better condition of older people compared to younger ones.

Due to the cross-sectional design of the study, it was not possible to measure a change over time of lifestyle habits. Only self-reported information about a general increase or decrease in habits, as perceived by the respondents, was available.

Questions concerning mental well-being and sleeping disorders refer to what happened in the 14 days before the interview. Therefore, we can only explore the recent mental health status without knowing whether it has improved or worsened compared to a baseline.

Lastly, the four domains of the SPAN questionnaire were used at individual level to evaluate single emotions, although the questionnaire was built to screen for PTSD symptoms using its total score. Future studies should also consider evaluating the difficulties in emotional regulation.

The major strength of this work is its rapid implementation and the involvement of a large sample of citizens, allowing to gain, in a period of emergency, valuable information for identifying vulnerable population subgroups.

## Conclusions

The restrictions imposed during the acute phase of the COVID-19 pandemic limited the daily life at different levels, leading to relevant emotional experiences in the sample, mainly anger and nervousness. These emotions may play an adaptive role in coping with the pandemic stressor, preparing the body and the mind to react. However, especially if persistent, they may simultaneously represent a form of post-traumatic stress reaction, particularly intense for female, younger and single persons. Anyhow, more longitudinal studies are required to better characterize the long-term impact of these reactions. Finally, our results claim that great attention should be reserved for lifestyle care, increasing the level of physical activity, reducing the consumption of alcohol and preserving a good amount and quality of sleep. This may be a compelling way to help people to better cope with the restrictions imposed by the COVID-19 pandemic and may inform public health programs on which behaviours address their efforts to reduce its psychological impact.

## Supplementary Information

**Additional file 1.**

## Data Availability

The datasets generated and analysed during the current study are not publicly available due to the necessity to protect data which are still under analysis for further studies, but are available from the corresponding author on reasonable request.
