# Supplementary material for: The emotional side of post-traumatic stress reaction during COVID-19 pandemic: an Italian survey
Source: BMC Public Health. 2022 Feb 16;22:333. doi: 10.1186/s12889-022-12749-1 (PMC8851796; doi:10.1186/s12889-022-12749-1)
Supplement: Supplementary file 1 — Additional file 1. [file 12889_2022_12749_MOESM1_ESM.pdf]

Survey regarding the impact of Covid-19 pandemic on lifestyles

The restrictive and isolation measures imposed by the Italian government to face and contain the spread of the Covid-19 virus could have an impact on the lifestyles of the population.

The Epidemiology Screening unit of the Reference Centre for Epidemiology and Cancer Prevention in Piedmont (CPO - AOU Città della Salute e della Scienza - Turin) is conducting an anonymous survey to understand if and to what extent these measures affect some behaviour of citizens. This information will make it possible to detect any problematic situations and plan targeted health promotion interventions together with actions to enhance some health services.

We invite you to answer a short questionnaire, which will take no more than 10 minutes to complete.

All data collected will be treated anonymously and reported in aggregate form. The results of this survey will be published in information and divulgation media and in scientific journals.

For any request for information, you can contact us at the email address indaginecovid19@cpo.it or by phone 011-6333873, 011-6333858 from Monday to Friday from 10.00 to 15.00.

We thank you for your precious collaboration.

The promoters of the initiative,

Working group of the S.S.D Epidemiology Screening Unit
AOU City of Health and Science of Turin

1. Sociodemographic features

What is your age? __ __

What is your gender?

◯ Male
◯ Female

What is your province of domicile / residence?

Curtain with all the Italian provinces

How many inhabitants does the city in which you live have?

◯ Up to 5,000 inhabitants
◯ Between 5,000 and 10,000 inhabitants
◯ Between 10,000 and 50,000 inhabitants
◯ Between 50,000 and 100,000 inhabitants
◯ Between 100,000 and 1,000,000 inhabitants
◯ Over 1,000,000 inhabitants

What is your current marital status?

◯ Married or cohabiting
◯ Single
◯ Widower
◯ Separated or divorced

What is your educational qualification?

◯ No title
◯ Elementary or lower secondary school
◯ High school diploma
◯ University degree or higher qualification

How many people (cohabitants, you excluded) make up your family unit at the time of completing this questionnaire? ___

(0 if your family is composed only of you)

How many people do you currently care for?

Minors (less than 14 years old) ___
Elderly (over 64 years old) ___
Disabled ___

How many rooms does the house where you live have?

- ◯ 1
- ◯ 2
- ◯ 3+

Does the house you live in have an outdoor space such as a garden or a terrace?

- ◯ Yes
- ◯ No

You were born in:

- ◯ Italy
- ◯ Europe
- ◯ North America
- ◯ Central and South America
- ◯ Africa
- ◯ Asia
- ◯ Oceania

If you were not born in Italy, please specify your country of birth: ___________________________

2. Information on work

What is your profession?

- ◯ Employee
- ◯ Freelance / self-employed
- ◯ Retired
- ◯ Housewife
- ◯ Student
- ◯ Unemployed

Your job is:

- ◯ full time
- ◯ part-time

How would you define your work activity with respect to the type of physical activity it involves? Please indicate which of these 4 categories corresponds best to the work you do / did

◯ Sedentary work - spend most of the time sitting (e.g. office employee, driver)

◯ Standing work - spend most of the time standing or walking, but intense effort is not required (e.g. representative, waitress, cook, kindergarten teacher, shop assistant, hairdresser, etc.)

◯ Manual work - medium physical effort is required such as handling heavy tools (e.g. ironer, weaver, domestic worker, plumber, mechanic, etc)

◯ Heavy manual work - vigorous physical effort is required such as handling very heavy tools (e.g. cleaning worker, farmer, professional athlete, metal worker, bricklayer, etc)

After the approval of the #Istayathome decree, did you continue / are you continuing to attend your usual workplace?

◯ Yes, nothing has changed since before

◯ Yes, but I have reduced attendance (I work both from home and at workplace)

◯ No, I only worked from home (agile / smart working)

◯ No, my employment is suspended

◯ I was fired

3. Physical activity in free time

In answering the following questions, refer to the period following the approval of the #Istayathome decree

1) Did you practice moderate physical activity (slight increase in breathing and heart rate or a little sweating)?

◯ No

◯ Yes

If so, on average how many hours a week did you spend on it? __ __

In what way?

Response with drop-down menu with: video tutorial on the internet, App for training, live tutorial, self-training, other (specify)

2) Did you practice intense physical activity (large increase in breathing and heart rate or profuse sweating)?

◯ No

◯ Yes

If so, on average how many hours a week did you spend on it? __ __

In what way?

Response with drop-down menu with: video tutorial on the internet, App for training, live tutorial, self-training, other (specify)

3) Has the time you dedicated to physical activity changed significantly after the approval of the #Istayathome decree?

◯ No
◯ Yes, it was reduced
◯ Yes, it was increased

If you have REDUCED or INCREASED the time devoted to physical activity please indicate why:

◯ Reasons related to the containment measures of Covid-19 infection
◯ Health reasons (specify) ____
◯ Other (specify) ____

4) How many hours a day did you spend on housework?

◯ Less than 1 hour ◯ 1-2 hours ◯ 3–4 hours ◯ 5-6 hours ◯ 7 hours or more

5) Has the time you spent on housework varied significantly?

◯ No
◯ Yes, it was reduced
◯ Yes, it was increased

If you have REDUCED or INCREASED the time devoted to housework, please indicate why:

◯ Reasons related to the containment measures of Covid-19 infection
◯ Health reasons (specify) ____
◯ Other (specify) ____

4. Eating habits

In answering the following questions, refer to the period following the approval of the #Istayathome decree. Consider all meals (breakfast, lunch, dinner and snacks) and remember to always answer, even when the answer is NEVER.

How many times have you eaten whole grains such as wheat, spelled, barley, brown rice, buckwheat?

Dropdown response with never, less than once a week, 1-3 times a week, 4-7 times a week, 8 or more times a week

How many times have you eaten vegetable / legume / puree soup (with or without some pasta or rice)?

Dropdown response with never, less than once a week, 1-3 times a week, 4-7 times a week, 8 or more times a week

How many times have you eaten red meat from beef (including veal), sheep, pork, horse?

Dropdown response with never, less than once a week, 1-3 times a week, 4-7 times a week, 8 or more times a week

How many times have you eaten cold cuts?

Dropdown response with never, less than once a week, 1-3 times a week, 4-7 times a week, 8 or more times a week

How many times have you eaten cheese?

Dropdown response with never, less than once a week, 1-3 times a week, 4-7 times a week, 8 or more times a week

How many times have you eaten legumes such as beans, lentils, chickpeas, broad beans, peas, soy (both fresh, dried, frozen and canned)?
In answering you do not consider the legumes consumed in first courses and soups.

Dropdown response with never, less than once a week, 1-3 times a week, 4-7 times a week, 8 or more times a week

How many times have you eaten cooked, raw or leafy vegetables?

Dropdown response with never, less than once a week, 1-3 times a week, 4-7 times a week, 8 or more times a week

How many times have you eaten salty snacks (e.g. pretzels, croutons, bag fries)?

Dropdown response with never, less than once a week, 1-3 times a week, 4-7 times a week, 8 or more times a week

How many servings of fresh fruit have you eaten? (for example one portion corresponds to an apple or a pear or a bowl of strawberries or three clementines or two kiwis or three apricots)

Dropdown response with none, less than 1 per week, 1-3 per week, 4-7 per week, 8 or + per week

How many servings of sweets or pastries have you eaten?

Dropdown response with none, less than 1 per week, 1-3 per week, 4-7 per week, 8 or + per week

How many brioches, croissants or snacks have you eaten?

Dropdown response with none, less than 1 per week, 1-3 per week, 4-7 per week, 8 or + per week

Normally, how many SPOONS or sachets of sugar (both white and brown) or SPOONS of honey have you used?

Dropdown response with none, less than 1 per week, 1-3 per week, 4-7 per week, 8 or + per week

How many glasses (125 ml) of wine did you consume?

Dropdown response with none, less than 1 per week, 1-3 per week, 4-7 per week, 8 or + per week

How many cans or bottles (330ml) of beer have you consumed?

Dropdown response with none, less than 1 per week, 1-3 per week, 4-7 per week, 8 or + per week

How many small glasses (40 ml) of spirits, bitters, liqueurs (e.g. whiskey, vodka, brandy, rum, grappa) did you consume?

Dropdown response with none, less than 1 per week, 1-3 per week, 4-7 per week, 8 or + per week

How many glasses or cans of carbonated and / or sugary soft drinks did you consume?

Dropdown response with none, less than 1 per week, 1-3 per week, 4-7 per week, 8 or + per week

Did you significantly change your eating habits after the #Istayathome decree was approved?

◯ No

◯ Yes

If so, for what reason?

◯ For reasons related to home isolation for the containment of Covid-19 infection
◯ To lose weight
◯ For health reasons (specify) ____
◯ Other (specify) ____

If so, how has the consumption of the following foods varied?

	Increased	*Unchanged*	*Decreased*
**Sausages**	◯	◯	◯
**Red meat**	◯	◯	◯
**Vegetables**	◯	◯	◯
**Fruit**	◯	◯	◯
**Sweets**	◯	◯	◯
**Alcohol**	◯	◯	◯
**Legumes**	◯	◯	◯
**Whole grains**	◯	◯	◯

Can you tell me your height? __ __ __ cm

Can you tell me your weight? __ __ __ kg

5. Smoking habits

Do you usually smoke traditional cigarettes / cigars / pipes or electronic cigarettes?

◯ I have never smoked

◯ I have smoked in the past
How many cigarettes (cigars, pipes) did you smoke per day? __ __
How many times a day did you smoke e-cigarettes __ __
At what age did you start smoking? __ __
At what age did you stop? __ __

◯ I smoke occasionally
How many cigarettes (cigars, pipes /) do you smoke per month? __ __
How many times a month do you smoke e-cigarettes? __ __

◯ I smoke habitually
How many cigarettes (cigars, pipes) do you smoke per day? __ __
How many times a day do you smoke electronic cigarettes __ __
At what age did you start smoking? __ __

If you currently smoke traditional cigarettes / cigars / pipes, has the number smoked per day changed since the #Istayathome decree was approved?

◯ No
◯ Yes, I reduced - How many cigarettes per day? __ __
◯ Yes, I increased - How many cigarettes per day? __ __

If you have REDUCED or INCREASED the number of cigarettes / cigars / pipes smoked per day for what reason:

◯ For reasons related to home isolation for the containment of Covid-19 infection
◯ Health reasons (specify) ____
◯ Other (specify) ____

If you currently smoke electronic cigarettes, has the number of times you used it per day changed since the #Istayathome decree was approved?

◯ No
◯ Yes, I have reduced - How many times a day? __ __
◯ Yes, I increased - How many times a day? __ __

If you have REDUCED or INCREASED the number of times per day you smoked e-cigarettes for what reason:

◯ For reasons related to home isolation for the containment of Covid-19 infection

◯ Health reasons (specify) ____

◯ Other (specify) ____

6. Health status

To date, have you received a diagnosis of Covid-19 infection confirmed by a swab?

◯ No

◯ Yes, without hospitalization

◯ Yes, with hospitalization

Did you receive a recommendation from a doctor to remain in isolation for possible Covid-19 infection?

◯ No

◯ Yes, because I had suspected symptoms of Covid-19 infection

◯ Yes, because I came into contact with positive people at Covid-19

Are you a healthcare professional?

◯ No

◯ Yes

Has a doctor ever diagnosed you with one or more of the following conditions or diseases?

High pressure

◯ No

◯ Yes - At what age? __ __ - Are you on drug therapy? ◯ No ◯ Yes

High cholesterol

◯ No

◯ Yes - At what age? __ __ - Are you on drug therapy? ◯ No ◯ Yes

High triglycerides

◯ No

◯ Yes - At what age? __ __ - Are you on drug therapy? ◯ No ◯ Yes

Diabetes

◯ No

◯ Yes - At what age? __ __ - Are you on drug therapy? ◯ No ◯ Yes

Malignant cancer

◯ No

◯ Yes - At what age? __ __ In which location? ____- Are you on drug therapy? ◯ No ◯ Yes

In addition to any previous pathologies, do you regularly use medications prescribed by a doctor? (for women, exclude contraceptives)

◯ No

◯ Yes

7. Mental well-being

When answering the questions below, please refer to the period which includes the last 14 days.

In the past two weeks, how many days have you had little interest or little pleasure in doing things?

◯ never

◯ a few days

◯ more than half of the days

◯ almost every day

In the past two weeks, how many days have you been feeling low, depressed or hopeless?

◯ never

◯ a few days

◯ more than half of the days

◯ almost every day

In the past two weeks, how much and with what intensity have you experienced restless or disturbed sleep?

◯ not at all

◯ a little

◯ moderately

◯ a lot

◯ very much

In the following questions indicate the FREQUENCY of the event and if the answer is different from "NEVER" fill in the second column - INTENSITY

Have you felt nervous or easily frightened in the past two weeks?

FREQUENCY	INTENSITY
◯ never<br>◯ only once<br>◯ 2-3 times<br>◯ 4 or more times, most days	◯ minimal discomfort<br>◯ moderate discomfort<br>◯ high discomfort<br>◯ extreme discomfort

In the past two weeks, have you felt irritable or have you had outbursts of anger?

FREQUENCY	INTENSITY
◯ never<br>◯ only once<br>◯ 2-3 times<br>◯ 4 or more times, most days	◯ minimal discomfort<br>◯ moderate discomfort<br>◯ high discomfort<br>◯ extreme discomfort

In the past two weeks, have you felt unable to experience feelings of sadness or affection?

FREQUENCY	INTENSITY
◯ never<br>◯ only once<br>◯ 2-3 times<br>◯ 4 or more times, most days	◯ minimal discomfort<br>◯ moderate discomfort<br>◯ high discomfort<br>◯ extreme discomfort

In the last two weeks, have you had any physical disturbances related to thoughts on the ongoing emergency? (eg, sweating, tremors, rapid heartbeat, shortness of breath, nausea, or diarrhea)

FREQUENCY	INTENSITY
◯ never<br>◯ only once<br>◯ 2-3 times<br>◯ 4 or more times, most days	◯ minimal discomfort<br>◯ moderate discomfort<br>◯ high discomfort<br>◯ extreme discomfort

In the last couple of weeks, have you felt that you are supporting / helping anyone?

◯ not at all
◯ a little
◯ enough
◯ a lot
◯ very much

In the last two weeks, how much have you been able to count on the support / help of these people?
multiple choice (answer all)

Family members or other relatives.	◯ always ◯ often ◯ sometimes ◯ never
Friends / acquaintances / neighbours.	◯ always ◯ often ◯ sometimes ◯ never
Other people free of charge (e.g. volunteers from associations)	◯ always ◯ often ◯ sometimes ◯ never
Other paid people (e.g. caregiver, housekeeper)	◯ always ◯ often ◯ sometimes ◯ never

Thanks for your attention and availability!
Your contribution was very valuable!
